# Bioactive Dressing: A New Algorithm in Wound Healing

**DOI:** 10.3390/jcm13092488

**Published:** 2024-04-24

**Authors:** Gianmarco Polverino, Francesca Russo, Francesco D’Andrea

**Affiliations:** Department of Plastic and Reconstructive Surgery, Federico II University of Naples, Via Pansini 5, 80131 Naples, Italy; francesco.dandrea2@unina.it

**Keywords:** wound healing, wounds, inflammation, angiogenesis, bioactive dressing, skin, stem cells, plastic surgery

## Abstract

Wound management presents a significant global challenge, necessitating a comprehensive understanding of wound care products and clinical expertise in selecting dressings. Bioactive dressings (BD) represent a diverse category of dressings, capable of influencing wound healing through various mechanisms. These dressings, including honey, hyaluronic acid, collagen, alginates, and polymers enriched with polyhexamethylene biguanide, chitin, and chitosan derivatives, create a conducive environment for healing, promoting moisture balance, pH regulation, oxygen permeability, and fluid management. Interactive dressings further enhance targeted action by serving as substrates for bioactive agents. The continuous evolution of BDs, with new products introduced annually, underscores the need for updated knowledge in wound care. To facilitate dressing selection, a practical algorithm considers wound exudate, infection probability, and bleeding, guiding clinicians through the process. This algorithm aims to optimize wound care by ensuring the appropriate selection of BDs tailored to individual patient needs, ultimately improving outcomes in wound management.

## 1. Introduction

Wound management poses a significant and growing challenge globally. Two key components for a proper approach to wound management are knowledge of wound care products and clinical experience in selecting dressings, both of which are essential to ensure evidence-based care. Optimal wound management requires accurate patient assessment, precise wound diagnosis, and a thorough understanding by healthcare professionals of the wound healing process, as well as the characteristics of dressings, including advantages and disadvantages, indications, and contraindications. This also involves a careful understanding of the properties of first-line interactive/bioactive dressing groups commonly used in clinical practice [1].

Bioactive dressings (BD) remain a topic of discussion within the medical community. They are characterized as dressings made from natural or synthetic materials that have the ability to impact the wound-healing process either directly or indirectly [2].

Their action can manifest through the release of bioactive factors or due to the presence of materials with endogenous activity. This category includes various types of products such as alginates, collagen (CG), hydrocolloids, biotextiles, chitosan, chitin, and their derivatives [3].

Interactive dressings have the potential to act as a platform for bioactive agents, enabling a more precise and tailored approach to address the unique conditions of ulcers and the specific requirements for skin restoration [4]. Key characteristics shared by BDs typically involve:

- Maintenance of a moist wound environment;
- Assistance in restoring normal pH levels;
- Permeability to oxygen;
- Promotion of optimal oxygen concentration within the ulcer site;
- Effective management of fluids;
- Biodegradability;
- Compatibility with biological systems;
- Antioxidant properties;
- Frequently necessitating secondary dressing application.

Schematically, BDs can be divided into two subgroups [5] (Table 1):

(1) Bioactive;
(2) Drug-loaded wound dressings.

**Table 1:** Types of Bioactive dressings.

(1) Bioactive Dressings	(2) Drug-Loaded Wound Dressings
Honey	Silver
Hyaluronic Acid	Iodopovidone
Collagen	Ozonides
Alginate	Mesoglycan
Chitin	DNA and ribosomes
Chitosan	Rigenase
Polymers enriched with PHMB (Polyhexamethylene Biguanide)	Matrix metalloproteinase inhibitors (MMPs inhibitors)

The number of BDs is destined to increase even further, thanks to advancements in new technologies. The objective is to develop the “optimal dressing” that meets specific requirements, including maintaining a moist environment, promoting epidermal migration, promoting angiogenesis and the synthesis of connective tissue, facilitating gas and nutrient exchange, protecting against bacterial infections, and being sterilizable, non-toxic, biodegradable, and hypoallergenic [6].

## 2. Bioactive Dressings

### 2.1. Bioactive Dressing: Honey

Honey, being a natural product containing enzymes, phenols, and sugars with antioxidant, anti-inflammatory, and antibacterial properties, consists of water, sucrose, glucose, fructose, amino acids, beeswax, pollen, pigments, minerals, and glucose oxidase. Honey can have various positive effects on the wound healing process, including suppressing inflammation, encouraging angiogenesis and immune response, and speeding up dermal repair and re-epithelialization [7].

#### 2.1.1. Anti-Inflammatory Activity

This activity is crucial for wound healing and an inadequate response can lead to delayed healing. Honey exhibits its anti-inflammatory activity through various mechanisms. Firstly, reducing the production of free radicals through its antioxidant properties, thereby limiting tissue necrosis [7]. Additionally, in vivo and in vitro studies have shown that honey reduces prostaglandin synthesis by inhibiting the activity of cyclooxygenase 1 (COX1) and cyclooxygenase 2 (COX2). [8]. A decrease in prostaglandins results in an improvement in edema, pain, and inflammation. Finally, honey blocks the expression of tumor necrosis factor-alpha (TNF-α) and reduces pro-inflammatory cytokine levels by modulating nuclear factor kappa B (NF-kB) levels. Finally, honey improves microcirculation and tissue oxygenation. This promotes tissue repair and tissue growth [9].

#### 2.1.2. Antioxidant Activity

Various components of honey are responsible for antioxidant activity such as flavonoids, phenolic acids, ascorbic acid, tocopherols, and some enzymes like superoxide dismutase and catalase [10].

#### 2.1.3. Antibacterial Activity

Honey acts directly on bacterial growth and survival due to its high acidity and osmolarity, through hydrogen peroxide and phenolic compounds, and indirectly promotes the production of lymphocytes, antibodies, cytokines, and nitric oxide. Both in vivo and in vitro studies also demonstrate the effectiveness of honey against antibiotic-resistant bacteria. It inhibits the formation of bacterial biofilm by reducing the expression of the gene related to its development and decreasing the metabolic activity of already formed biofilm [11,12,13,14,15,16,17,18,19].

#### 2.1.4. Neo-Angiogenic Activity

It has been widely demonstrated that honey promotes neoangiogenesis and the proliferation of endothelial cells thanks to the hydrogen peroxide produced by the glucose oxidase enzyme present in honey. Activation of this enzyme stimulates the production of growth factors, including vascular endothelial growth factor (VEGF), by macrophages [20].

VEGF is a key molecule involved in the formation of new blood vessels, known as neoangiogenesis [21].

This process is crucial for wound healing and tissue repair as it increases the availability of oxygen and nutrients at the site of interest. Furthermore, the high number of sugars in honey further stimulates the neoangiogenesis process. In summary, honey acts synergistically through hydrogen peroxide and nutrient supply to promote neo-angiogenesis and the proliferation of endothelial cells.

#### 2.1.5. Side Effects

Honey, despite being a natural product, faces limitations in its application. Its composition is highly variable, primarily influenced by botanical origin and geographical location, and not all of its components have well-understood biological activities. Collection, processing, and treatment methods can also impact its characteristics. These alterations affect bioactivity profiles and, consequently, therapeutic efficacy. Medical-grade honey undergoes sterilization through gamma irradiation to eliminate potential *Clostridium* spores. They are produced under strict hygiene standards, without contaminants or pesticides in their composition. Manuka honey is among the most widely utilized honey in the medical field, originating as a monofloral honey from New Zealand and Australia. Bees primarily collect nectar and pollen from *Leptospermum scoparium* (known locally as manuka) to produce it. Apart from manuka, other honeys like tualang, kanuka, and capilan also display notable antibacterial effects, although these may be diminished by high-temperature heat treatments [22,23]. Considering the presence of various substances with antibacterial properties, such as methylglyoxal, leptosperine, or methyl syringate, the concept of UMF (unique manuka factor), also known as “non-peroxide-dependent activity” (NPA), has been devised. For instance, honey labeled UMF 5 possesses the same antibacterial activity as a 5% phenol solution. The rating can range from UMF 5+ to UMF 20+; the higher the value, the greater the antibacterial activity of the product.

#### 2.1.6. Dressings

Honey-based dressings can be employed for treating both acute and chronic wounds, with certain limitations in the case of burns. They prove particularly beneficial in the presence of critical bacterial colonization or when bacterial strains do not respond to conventional antibiotic therapy [11]. These dressings are mainly used on wounds with a mild to moderate level of exudate to prevent excessive dilution of honey caused by the wound exudate [24]. Research also indicates the potential use of honey dressings on highly exudative wounds in the case of infection. Notably, even when honey is considerably diluted by the wound exudate, it maintains sufficiently potent antibacterial activity to inhibit bacterial growth (with minimal inhibitory concentration values below 11%) [11]. These dressings are commercially available in various forms, including creams, ointments, and impregnated gauzes as simple dressing options, while hydrogels, alginates, and pads are categorized as advanced dressings. It is crucial to note that honey dressings always necessitate the application of a secondary dressing and can be utilized under compression bandaging.

### 2.2. Bioactive Dressing: Hyaluronic Acid

Hyaluronic acid (HA) is a natural polymer belonging to the glycosaminoglycans (GAGs), a heterogeneous group of polysaccharides. It is mainly present in the vitreous humor, joints, umbilical cord, skin, and connective tissue. Nowadays, it is primarily obtained through bacterial fermentation.

HA is composed of disaccharide units of β-D-glucuronic acid and N-acetyl-D-glucosamine linked by β-1,3 and β-1,4 glycosidic bonds. It has a semi-rigid structure [25], with a variable molecular weight. Its formula is characterized by a high number of carboxylic (-COOH) and hydroxyl (-OH) groups, providing pronounced hydrophilicity. At physiological pH, the carboxylic and acetoamide groups on the molecule’s surface form hydrogen bonds with water, stabilizing the biopolymer’s secondary structure. High molecular weight HA (HMW-HA) exhibits greater stability and viscoelasticity than smaller molecules. It is crucial to note that the rheological properties of HA depend not only on molecular weight but also on the ionic charge of the solution (ionic strength), pH, and temperature. For instance, in case of skin damage, the wound bed’s pH is around 8 and reaches a value of 5 at the end of the healing process [26].

Under normal conditions, HA is mainly synthesized as HMW-HA. When the extracellular matrix (ECM) homeostasis is disrupted by pathological conditions, endogenous HMW-HA can be degraded more rapidly by hyaluronidases (HYAL) and reactive oxygen species (ROS). This imbalance leads to a higher concentration of low molecular weight HA (LMW), which can be further fragmented into shorter oligomers (o-HA). These changes underlie its multiple actions in the wound-healing process [27], as follows:

- Fibrin plug formation;
- Production and release of interleukins;
- Stimulates the invasion and proliferation of fibroblasts along with fibronectin and induces the formation of myofibroblasts, essential in the wound contraction process;
- Stimulates the migration and proliferation of keratinocytes.

#### Dressings

HA dressings should be used on acute and chronic cleansed wounds, with low to medium exudate. They require a secondary dressing.

We can schematically divide them into [28]

- Simple dressings: gauze, creams, and sprays, sometimes enriched with sulfadiazine, silver, and collagenase;
- Vials for local infiltration or topical treatment (HA with polynucleotides);
- Granules and powders;
- Esters Hyaff-11;
- Sponges with HA and native equine type 1.

The Esters Hyaff-11 family [29], consisting of semi-synthetic biopolymers, enables the production of various biomaterials through the esterification of the carboxyl groups of the HA molecule with benzyl alcohol. Esterification protects the molecule from rapid enzymatic degradation, allowing a longer in situ presence of the device. Upon contact with wound exudate, it transforms into a hydrophilic gel that coats the wound, creating a HA-rich interface that provides the ideal moist environment for wound healing.

### 2.3. Bioactive Dressing: Collagen

CG is the primary protein found in the ECM of the human body. It is synthesized by fibroblasts and plays a crucial role in various stages of the healing process. Its chemotactic effect on macrophages and fibroblasts, the predominant cells during the inflammation, proliferation, and maturation phases of wounds, significantly contributes to this process. There are 29 types of CG, divided into fibrillar and non-fibrillar categories. Among the fibrillar types, the most relevant ones in the skin are types I, II, and V, while among the non-fibrillar types, types IV and XVIII stand out [30].

#### 2.3.1. Extracellular Matrix Remodeling

The remodeling phase is essential in the healing of cutaneous wounds, marked by an active reorganization of the ECM. During this phase, there is an increase in crosslinking between CG fibers, leading to scar maturation. The formation of scar tissue replaces damaged tissue with CG-rich type I connective tissue, characterized by densely grouped and oriented CG fibrils. The degree of fibrosis varies between anatomical sites and individuals, influenced by factors such as mechanical tension and genetic predisposition. The inflammatory response plays a key role in scar formation, affecting the abnormal interaction between keratinocytes and fibroblasts, fibroblast–myofibroblast transformation, and excessive ECM deposition, resulting in various types of scars. A detailed understanding of these processes is crucial for the development of targeted therapies, with a specific emphasis on regulating remodeling processes, including CG synthesis and degradation mechanisms [31].

#### 2.3.2. Anti-Inflammatory Activity

Following tissue damage, CG fibers in the ECM activate platelets, leading to their aggregation and the formation of the platelet plug. CG types I and IV play a crucial role as mediators of anti-inflammatory activity, acting as a chemotactic agent for neutrophils, promoting phagocytosis, influencing the immune response, and modulating gene expression [32]. The presence of a CG matrix accelerates the inflammatory response, contributing to a rapid restitution ad integrum. Reduced CG levels in chronic wounds are associated with delayed healing [33].

#### 2.3.3. Neo-Angiogenic Activity

CG type I promotes angiogenesis through the release of C peptide and attracting endothelial cells. Conversely, CG type IV and XVIII (non-fibrillar) exert anti-angiogenic actions, as their proteolytic fragments hinder the migration and growth of endothelial cells, leading to apoptosis [34,35].

#### 2.3.4. Dressing

Wound dressings comprising 100% non-hydrolyzed CG are employed for treating both burns and wounds [36]. These dressings, which are typically non-porous and chemically similar to the CG ECM, are suitable for non-infected wounds that require intermediate exudation. This prevents excessive dryness associated with the rapid evaporation of exudate. Moreover, the CG pad acts as a scaffold, expediting wound healing by facilitating the presence of fibroblasts, macrophages, and neutrophils. In some cases, these described pads are combined with Manuka honey, known for its ability to reduce wound pH, inhibit matrix metalloproteinases (MMPs), and exhibit additional antimicrobial action [37].

Another available formulation involves triple-helical CG associated with HA. This dressing utilizes not only the characteristics of CG but also the hydrating, proliferative, and migratory properties of HA, along with promoting angiogenesis. Formulations featuring hydrolyzed and denatured CG have been introduced to overcome limitations associated with triple-helical CG, including poor solubility and prolonged solubility in an aqueous environment [36].

Certain pads, in addition to non-hydrolyzed bovine CG, are linked to polypeptides and glycerin or oxidized regenerated cellulose (ORC), enabling greater absorbent capacity [38]. Formulations involving ORC appear capable of

(1) Reducing enzymatic activity on the wound bed, thereby promoting the deposition of new tissue;
(2) Enhancing the release of platelet-derived growth factor (PDGF).

A final formulation combines the capabilities of denatured CG with the absorbent properties of carboxymethyl cellulose and sodium alginate. It also includes ethylenediaminetetraacetic acid (EDTA), which binds calcium, regulating MMPs, and CGases [39].

### 2.4. Bioactive Dressing: Alginate

Alginate, derived from various macroalgae, is a polymer found in many coastal areas worldwide. Primarily extracted from brown algae, it can be rendered water-soluble for advanced BDs. Water-soluble alginate, such as sodium alginate, exhibits significant rheological properties like gelation, viscosification, and dispersion stabilization, dependent on its chemical structure of M-blocks (mannuronic) and G-blocks (guluronic). The high content of M-blocks is beneficial for chronic wound healing, stimulating cytokine production. Non-toxic and biocompatible alginate is a hydrophilic biopolymer suitable for various advanced clinical and biomedical applications [40].

#### 2.4.1. Anti-Inflammatory Activity

The results obtained from a study on a full-thickness excision wound model in rats indicate that the use of alginate dressings can enhance the natural wound-healing process while concurrently inhibiting cytokines associated with fibrosis. This results in a reduction in wound size and an increase in epithelial cell proliferation. These findings suggest that topical treatment with alginate for skin defects could be highly effective in reducing wound dimensions; further studies are needed to evaluate the precise mechanism of epithelial cell proliferation induced by alginate treatment [41]. Additionally, a pronounced reduction in TNF-α in solution was observed with the use of alginate. All tested wound dressings demonstrated a high binding affinity, allowing only negligible amounts of cytokines to be subsequently released [42].

#### 2.4.2. Antimicrobial Activity

Tests on the antimicrobial activity of alginate-based wound dressings have confirmed their ability to effectively inhibit the growth of both Gram-positive and Gram-negative bacteria. No bacterial growth was observed after a 24-h treatment with these dressings for *P. aeruginosa*, *K. pneumoniae*, and *E. coli.* Silver-containing alginates demonstrated effective inhibition of *S. aureus* growth, while alginate alone still achieved a strong inhibitory effect. In the case of *C. albicans*, only dressings with alginate and nano-Ag achieved a complete reduction of viable yeast cells. Alginate + ionic Ag formulations showed a significant reduction in microbial growth, while alginate alone exhibited a modest inhibition.

In conclusion, alginate-based wound dressings have demonstrated significant and swift antimicrobial activity against a range of tested microorganisms, suggesting their potential for effective wound management [42].

#### 2.4.3. Dressing

Alginate dressings display several fascinating properties such as high biocompatibility, low toxicity, cost-effectiveness [43], and hemostatic properties [44].

They can take various forms, including hydrogels, films, nanofibers, and wafers. Hydrogels, films, and nanofibers containing sodium alginate demonstrate significant beneficial properties in the context of wound dressings. Hydrogels, through the use of nanoparticles and bioactive agents, exert a positive influence on wound healing by enhancing antibacterial activity and promoting cell proliferation. Films, appropriately enriched with antibacterial agents, exhibit high activity against bacteria and accelerate re-epithelialization in vivo. Nanofibers surpass commercial dressings in reducing cytokine production and significantly improving wound healing by facilitating re-epithelialization, vascularization, and hair follicle formation. The topical application of formulations with sodium alginate has shown a significant reduction in wound area in vivo. Overall, alginate-based materials hold promise for the future development of advanced dressings for effective wound management [45].

### 2.5. Bioactive Dressing: Chitin and Chitosan

Chitosan is a polymer derived from the deacetylation of chitin, a fundamental component of the exoskeleton in various crustaceans such as lobsters, crabs, and shrimp. It exhibits antimicrobial activity and has proven to be an effective material for wound dressing. Chitosan is insoluble at neutral or basic pH due to its free amino groups, while at acidic pH, it becomes soluble in water due to the protonation of amino groups [46,47]. It is a biocompatible molecule that stimulates hemostasis and accelerates tissue regeneration, attributed to the presence of N-acetyl glucosamine within it [48,49].

Chitosan has demonstrated analgesic properties when applied topically to open wounds, such as burns, skin abrasions, cutaneous ulcers, and grafted areas [50]. Additionally, due to its cationic nature, chitosan exhibits antimicrobial action [51], inhibiting the growth of microorganisms like *Escherichia coli*, *Fusarium*, *Alternaria*, and *Helminthosporium* [52]. Chitosan also exerts anti-inflammatory effects by inhibiting prostaglandin E2 and cyclooxygenase-2, reducing proinflammatory cytokines and increasing the expression of the anti-inflammatory cytokine interleukin-10 [53]. It can be employed to create diverse types of wound dressings, including membranes, coatings, hydrogels, fibers, powders, and nanoparticles.

### 2.6. Bioactive Dressing: PHMB

Polyhexamethylene biguanide (PHMB) is a potent antimicrobial agent belonging to the biguanide family. The structure of PHMB is characterized by repeated biguanide groups linked by hexamethylene chains. Its lethal action primarily occurs through interaction with bacterial cytoplasmic membranes, causing immediate damage followed by diffusion and irreversible loss of essential cell components. This sensitivity to PHMB is studied in various *Escherichia coli* cultures. PHMB is a low-toxicity and safe disinfectant widely used in various clinical applications, including the disinfection of contact lenses and wound dressings [54].

#### 2.6.1. Antibacterial Activity

Research indicates that PHMB interacts solely with negatively charged phospholipid membranes. Moreover, it is only at concentrations surpassing a critical threshold that the adsorption of PHMB leads to a notable reduction in bilayer stability [55]. Even if some studies suggest that PHMB may traverse the membrane by forming salt bridges with anionic phospholipids, its predominant mechanism of action seems to be translocation across the cytoplasmic membrane. Intensive interactions between PHMB and the lipid bilayer, with possible stochastic fluctuations of the PHMB chain, suggest a translocation process through the lipid membrane. Regardless of the mechanism of entry into cells, PHMB shows extensive interaction with DNA, adopting a clustered conformation. This aggregation, especially with numerous PHMB chains, could interfere with DNA replication or activate repair processes. In conclusion, membrane disruption seems unlikely to be the primary mechanism of action for PHMB, supporting the recent hypothesis of translocation through the bacterial membrane and direct interaction with genetic material [56].

#### 2.6.2. Side Effects

Human skin fibroblasts (HSF) and human vascular endothelial cells were cultured in a culture medium supplemented with 10% fetal bovine serum. In two different plates, a PHMB hydrogel-modified wound scaffold dressing, with a diameter of 16 mm, and an unmodified wound scaffold dressing were applied. The percentage survival rate of cells treated with the PHMB hydrogel dressing and the dressing group without PHMB was overlapping [57]. In 2017, the Scientific Committee of Consumer Safety gave the opinion that the use of PHMB as a preservative in all cosmetic products up to 0.1% is safe [58].

#### 2.6.3. Dressing

Dressings containing PHMB are commercially available in the form of foam, sponge, and rolls. The antibacterial capacity of PHMB was assessed by evaluating its effectiveness in reducing bacterial load in a randomized controlled trial. The results highlighted that, although both achieved the goal of reducing bacterial load and pain, the wounds treated with PHMB exhibited a significantly greater decrease in bacterial presence compared to those treated with silver [59].

## 3. Drug-Loaded Wound Dressings

### 3.1. Drug-Loaded Wound Dressings: Silver

The issue of using topical antimicrobials in wounds is controversial due to concerns related to bacterial resistance. In this context, silver-based agents have attracted growing attention. Silver, known for its antimicrobial properties, has a long history of use in medicine. It has the ability to inactivate bacterial enzymes, block DNA synthesis, and disrupt bacterial membranes [60]. The response to silver nanoparticles varies among different biological systems, influenced by differences in surface properties and cell receptors. The toxic effects of silver can arise from both direct interactions, such as binding to membranes and receptors, and indirect effects, such as the activation of signaling pathways. Although low concentrations are recommended for clinical applications, toxicity can vary. At present, further systematic studies are essential for a comprehensive assessment of silver toxicity [61]. Silver is available in various formulations, packaged with practically every type of dressing, including creams, alginates, CG, hydrofibers, sponges, films, hydrogels, foams, and hydrocolloids.

### 3.2. Drug-Loaded Wound Dressings: Iodine

Iodine has been topically used for centuries to heal wounds [62]. Its antimicrobial action, discovered in 1882, is well-established and has provided a scientific basis for iodine-based products in the treatment of infected wounds [63]. To minimize side effects, iodine is commonly bound to carrier molecules like povidone-iodine and cadexomer iodine [64]. Iodine induces the oxidation of proteins, nucleotides, and fatty acids in microorganisms, leading to their cellular death [65,66]. It exhibits a broad antimicrobial spectrum, acting against Gram-positive and Gram-negative bacteria, antibiotic-resistant strains, fungi, protozoa, and viruses [67,68]. Iodine has demonstrated greater efficacy against mature biofilms compared to silver, suggesting potential clinical superiority [69]. The occurrence of adverse effects, including thyroid function deviation, has not been observed more frequently with iodine use than with other BDs [70]. Iodine is commercially available for wound treatment in the form of gel, foam, and dressings.

### 3.3. Drug-Loaded Wound Dressings: Ozonides

Ozone is formed in a reversible endothermic reaction catalyzed in the atmosphere by ultraviolet rays [71]. Ranked as the third most potent oxidizing agent after fluorine and persulfate, ozone is highly reactive and unstable, with a lifespan of about 3 s in the gas phase, making storage impractical [72].

The German chemist Schönbein, in 1840, identified ozone after a reaction between water and electricity. Subsequently, surgeon Erwin Payr extensively used ozonated oil as a therapy to treat infected wounds during World War I [73].

Ozonide-based wound care devices can be distinguished as spray oils, creams, impregnated gauze, oily preparations in pre-filled syringes, and cleansers [74,75]. They can be combined with hydrogels or alginates.

#### Antibacterial Mechanism and Anti-Inflammatory Activity

Ozone is recognized as a potent bactericidal, antiviral, and antifungal agent, empirically used for clinical therapeutic purposes in various conditions such as post-surgical fistulas, pre-secure ulcers, and chronic wounds like trophic ulcers, ischemic ulcers, diabetic wounds, psoriasis, and athlete’s foot. Ozone’s beneficial effects on wound healing are linked to reducing microbial infection, debridement effect, modulation of the inflammatory phase, stimulation of angiogenesis, and biological and enzymatic reactions promoting oxygen metabolism to enhance wound healing [76].

Ozonized oil demonstrates early response, increased cell involvement in the repair process, superior angiogenesis with elevated VEGF, and cyclin D1 expression when used on wounds [77].

A variety of ozonized vegetable oils have shown antibacterial effects against *S. aureus*, *E. faecalis*, *E. faecium*, *S. pyogenes*, *E. coli*, *P. aeruginosa*, and *Mycobacterium* spp. [78,79]. Most research focuses on sunflower oil and its commercial preparations. Ozonized olive oil has proven effective in in vivo studies in rats against *S. pyogenes* and *S. aureus* [80].

Daily use of ozonized oil on infected lesions in diabetic and atherosclerotic patients eliminates infections and promotes rapid healing [81]. Medical ozone treatment activates the antioxidant system and can serve as an alternative therapy for diabetic ulcers and their complications [82]. Ozonized oil may be an alternative for biofilm control in patients with prosthetic stomatitis [83]. Gum massage with ozonized oils proves to be an effective alternative against plaque-induced gingivitis [84]. Ozonized oil can suppress inflammation in atopic dermatitis, showing potential as a treatment for this condition. In acute skin wounds, ozonized oil significantly improves the healing process [85].

### 3.4. Drug-Loaded Wound Dressings: Mesoglycan

Mesoglycan (MSG) is a mixture of GAGs extracted from the intestinal mucosa of pigs. It has shown promise in wound healing by inducing migration and early differentiation of keratinocytes [86,87]. There is increasing evidence that GAGs play a role in reepithelialization of chronic skin ulcers, particularly in lower limb ulcers of venous and arterial origin. MSG has been demonstrated to promote the externalization of microvesicles by human keratinocytes in vitro [87]. It exhibits antithrombotic and pro-fibrinolytic actions, proving effective in treating vascular disorders and providing beneficial effects in patients with chronic venous ulcers and peripheral arterial diseases [88].

The commercially available device is primarily composed of MSG, alginate, and HA. The MSG in the dressing is a natural preparation of GAGs, including heparan sulfate, dermatan sulfate, slow-moving heparin, and chondroitin sulfate [89]. These dressings do not have an antibacterial effect. For this reason, they should be used exclusively on non-infected wounds. They are suitable for use on clean ulcers with moderate to low exudate or wounds in the granulation phase.

### 3.5. Drug-Loaded Wound Dressings: DNA and Ribosomes

Polydeoxyribonucleotide (PDRN) is a drug extracted from the gonads of trout, containing a mixture of polynucleotides [90,91]. It is a DNA fraction with a double-helix structure, comprising deoxyribonucleotides. Triggered by cellular enzymes, it supplies purines and pyrimidines [92]. PDRN induces cell growth via recovery metabolic pathways and adenosine A2a receptor activation, fostering inflammatory resolution. Moreover, it enhances VEGF, aiding endothelial cells in blood vessel formation and supporting granulation tissue development [93], and is able to promote the proliferation of human pre-adipocytes in vitro, affirming the potential use of PDRN for therapeutic and regenerative applications [94].

In a large double-blind randomized controlled trial (RCT), 216 diabetic patients with Wagner grade 1 or 2 ulcers were divided into two groups, with one receiving a placebo and the other PDRN for 8 weeks. Administered intramuscularly and perilesionally, PDRN demonstrated a twofold increase in the complete healing rate of challenging foot ulcers compared to the placebo within an 8-week period [91]. Additionally, a study on diabetic foot ulcers (Wagner grade 1–4) involving PDRN administration revealed improved tissue oxygenation, increased angiogenesis, and reduced inflammation after a two-week treatment [95]. Another randomized clinical trial on pressure ulcers highlighted the significant reduction in wound surface area with PDRN administration, demonstrating its efficacy without adverse effects during treatment [96].

PDRN is indicated for use on cleansed wounds. Topical formulations are accessible in prefilled syringes and cream presentations. Certain formulations may contain HA to enhance the formation of granulation tissue.

### 3.6. Drug-Loaded Wound Dressings: Rigenase

Rigenase^®^ is a new specific extract of *Triticum vulgare* (TVE), a plant belonging to the Graminaceae family, renowned for its hydrating properties that retain a scavenging effect against free radicals, demonstrating significant antioxidant activity. Additionally, it maximizes the tissue regeneration process through increased chemotaxis, fibroblastic proliferation, and maturation. These properties result from the enhanced synthesis of proteins, proline uptake, and the upregulation of crucial factors such as MMP-2, MMP-9, CG I, and elastin [97]. Widely used in conjunction with polyhexanide dressings, it treats skin lesions such as pressure ulcers, sores, burns, and delayed healing, requiring stimulation of the repair process. When applied as a cream or impregnated gauze, they form a protective layer against the external environment, creating favorable conditions for faster skin re-epithelialization and more effective wound healing [98].

Dressings containing Rigenase^®^ are suitable for application on both acute and chronic wounds that are cleansed, exhibiting low to medium exudation levels. These dressings are available in various forms, including impregnated gauze, cream, spray, or hydrogels [98,99,100].

### 3.7. Drug-Loaded Wound Dressings: MMPs Inhibitors

MMPs were first discovered in 1962 in the tadpole’s tail during frog metamorphosis. These enzymes are acknowledged as the key proteases involved in controlling the degradation of the ECM [101]. Beyond their role in remodeling tissues, MMPs also contribute to regulating various targets outside the matrix, including cell surface receptors, cell–cell adhesion molecules, cytokines, clotting factors, chemokines, and other proteinases [102]. Up to now, 23 MMPs have been recognized in the human body. MMPs are categorized into six classes based on their substrate specificity, primary structures, and cellular localization [103]:

- CGases (MMP-1, MMP-8, and MMP-13);
- Gelatinases (MMP-2 and MMP-9);
- Stromelysins (MMP-3, MMP-10, and MMP-11);
- Matrilisins (MMP-7 and MMP-26);
- Membrane-type MMPs (MMP-14, MMP-15, MMP-16, MMP-17, MMP-24, and MMP-25);
- Others (MMP-12, MMP-19, MMP-20, MMP-21, MMP-23, MMP-27, and MMP-28).

Their functions extend beyond ECM modifications, encompassing involvement in inflammation. MMP-8, released by neutrophils, is crucial for wound debridement and cleavage of damaged CG. The lack of MMP-8 can lead to healing delays, TGF-b signaling, and inflammation. Similarly, MMP-1 produced by keratinocytes bound to type I CG plays a key role in keratinocyte migration. MMP-2 and MMP-9 are involved in cell migration during wound healing, participating in laminin-5 proteolysis and contributing to re-epithelialization. In diabetic patients, wound healing requires a balance between CGous and non-CGous ECM components, with MMP-8 and MMP-9 degrading damaged CG and facilitating keratinocyte migration [104].

Dressings targeting MMPs can be broadly classified into two main groups [102]:

1. MMP inhibitors;
2. MMP modulators.

The distinction between the two lies in the fact that the former are specifically designed for their impact on proteases, while the latter act by modulating the ulcer microenvironment. Among devices inhibiting MMP secretion, two stand out:

1. ORC/CG Matrix: it is a sterile lyophilized pad composed of 55% CG and 45% ORC. This device reduces the activity of elastase, MMPs (particularly CGase and gelatinase), and oxygen-free radicals [105]. Interacting with the wound, it hinders tissue degradation and promotes granulation tissue synthesis through mechanisms such as reducing proteolytic activity and free radical damage, binding and stabilizing growth factors like PDGF, increasing the recruitment of macrophages and fibroblasts, and fostering fibroblast proliferation [106];
2. Lipid-Colloid Technology with Nano Oligosaccharide Factor (TLC-NOSF): this range of dressings consists of carboxymethylcellulose particles distributed in a vaseline network and impregnated with NOSF on a non-occlusive soft non-woven polyester layer. Upon contact with exudate, hydrocolloid particles form a gel that interacts with Vaseline by creating a lipidocolloid film, maintaining a moist environment in the wound, and reducing discomfort during dressing removal [107]. The potassium salt of sulfated oligosaccharides enhances the reparative process by inhibiting MMPs, interacting with growth factors, and restoring their biological functions [108].

Several devices on the market regulate ulcer bed characteristics (ES pH), leading to a reduction in MMP levels. These may include modulators of wound environment pH through ion exchange mechanisms, acetate mesh media containing potassium chloride, rubidium chloride, calcium chloride, zinc chloride, potassium citrate, and citric acid. Use is indicated in cleansed lesions, both acute and chronic.

## 4. A New Practical Algorithm in Approaching Challenging Wounds

In recent years, multiple BDs have been introduced to the market [5]. Some of the latest products were introduced in 2023 [98]. The BD market is continuously expanding, with the number of available dressings increasing each year. It becomes essential, for this reason, to stay updated on new products and understand their mechanisms of action to choose the most suitable BD. The high number of available dressings makes the selection process challenging for both novices and experienced individuals. For this reason, we have developed a practical algorithm based on every BD action as exposed in previous paragraphs and on authors’ everyday practice. Although other decision-making algorithms on advanced dressings have been published, some do not consider dry wounds [109], limiting their practical use, while others do not take into account the latest BDs [110,111,112].

**Figure 1:**
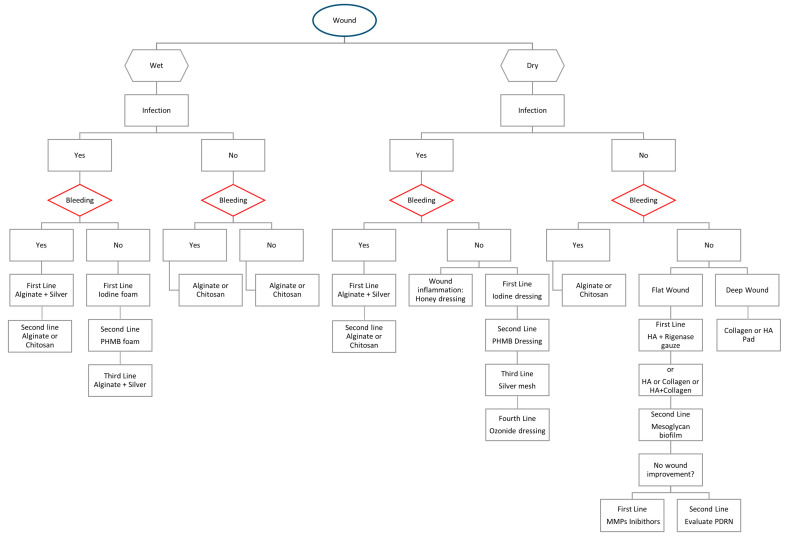
A new practical algorithm in approaching challenging wounds. PHMB: Polymers enriched with polyhexamethylene biguanide, HA: Hyaluronic acid, MMPs inhibitors: Matrix metalloproteinase inhibitors, PDRN: Polydeoxyribonucleotide.

Our algorithm differs from other published algorithms because it is based on the initial clinical assessment of wound exudate both wet or dry, the clinical evaluation of the probability of infection [113], and the presence of bleeding, leading step by step to the selection of the most suitable dressing. It also allows for the assessment of alternative BDs in case those considered first-line are not available as happens in everyday practice (Figure 1). In cases of infected non-bleeding wounds, absorbent dressings containing iodine are recommended as first-line treatment, followed by PHMB as second-line, and silver as third-line in decreasing order of antimicrobial potency [59,69,114]. They have to be absorbent as foams if there is exudate or not absorbent as gauze if there is a dry wound. In case of bleeding wounds, the use of alginate or chitosan is always recommended, with or without the addition of silver if infection is suspected [44,115]. When treating an inflamed wound, a honey-based dressing should be considered in the early stages [7,9]. Non-infected and dry ulcers should be treated primarily with HA (with or without Rigenase) or CG dressings of varying thickness depending on the depth of the wound [36,37,38,39,98,99,100]. As a second-line treatment, MSG may be used [89]. Only if there is no improvement should dressings containing MMP inhibitors be considered, which, unlike HA, CG, and MSG, have an indirect action on the ECM. The use of PDRN remains niche and we recommend it only for refractory ulcers, as the commercially available formulations require multiple daily applications for creams, making them impractical, or injections, which may be poorly tolerated by the patient [91,95,96].

We believe that this algorithm, based on the characteristics of all BDs mentioned in this article and our practical experience, can be beneficial in approaching complex wounds and choosing the most suitable BD.

## 5. Conclusions

BDs offer versatile and innovative solutions for wound management. Thanks to their ability to positively influence the healing process through diversified mechanisms, BDs represent a fundamental pillar in modern wound care. The continuous evolution of these products, along with their increasing availability in the market, complicates the selection of the most suitable dressing. By adopting an algorithm, it is possible to streamline the selection of the most appropriate bioactive dressing for adequate wound management.
